# Dapagliflozin in Heart Failure and Acute Myocardial Infarction: A Systematic Review of the Association in Diabetic Patients

**DOI:** 10.7759/cureus.65914

**Published:** 2024-08-01

**Authors:** Lina M Al-Tarawneh, Abedallah J Al-Adwan, Faisal A Al-shaikhly, Mazin M Almomani, Rahaf T Oduibat

**Affiliations:** 1 General Practice, Jordan University Hospital, Amman, JOR; 2 General Practice, Jordanian Royal Medical Services, Amman, JOR; 3 Medicine and Surgery, University of Jordan, Amman, JOR; 4 Medicine and Surgery, Jordan University Hospital, Amman, JOR

**Keywords:** cardioprotection, glycemic control, quality of life, hospitalization, sglt2 inhibitors, cardiovascular outcomes, type 2 diabetes mellitus, myocardial infarction, heart failure, dapagliflozin

## Abstract

This systematic review explores the impact of dapagliflozin on heart failure (HF) and acute myocardial infarction (MI) in patients with type 2 diabetes mellitus. By analyzing recent studies, including both randomized controlled trials (RCTs) and retrospective analyses, this review provides insights into the cardiovascular effects of this sodium-glucose cotransporter 2 (SGLT2) inhibitor. The findings consistently demonstrate the benefits of dapagliflozin in reducing HF-related hospitalizations and improving outcomes for patients with established HF. These positive effects appear to extend beyond glycemic control, suggesting multiple mechanisms of action. The impact of dapagliflozin on acute MI outcomes is less clear, with mixed results across studies. Importantly, dapagliflozin shows promise in improving the quality of life of patients and is generally well-tolerated. The review suggests that dapagliflozin may play a significant role in managing cardiovascular risk in diabetic patients, particularly those with or at risk of HF. While the evidence is encouraging, the review also highlights areas requiring further investigation. These include determining the patient subgroups most likely to benefit from dapagliflozin, elucidating the precise mechanisms underlying its cardioprotective effects, and carrying out long-term outcome studies.

## Introduction and background

Heart failure (HF) and myocardial infarction (MI) represent significant cardiovascular complications in patients with diabetes mellitus, contributing substantially to morbidity and mortality in this population [1,2]. The intricate relationship between diabetes and cardiovascular disease has been well-established, with diabetes not only increasing the risk of developing HF and MI but also worsening outcomes in patients who experience these events. HF in diabetic patients is characterized by a complex pathophysiology involving metabolic disturbances, structural changes in the myocardium, and altered cellular signaling pathways [2]. The diabetic heart is subject to increased oxidative stress, mitochondrial dysfunction, and impaired calcium handling, which collectively contribute to both systolic and diastolic dysfunction [3]. Moreover, diabetic patients are at a higher risk of developing coronary artery disease, which can lead to ischemic cardiomyopathy and acute coronary events. Acute MI in the setting of diabetes presents unique challenges. Diabetic patients often experience atypical symptoms, leading to delayed diagnosis and treatment. Furthermore, the diabetic milieu is associated with a prothrombotic state, impaired collateral vessel formation, and reduced myocardial salvage following reperfusion therapy [4]. These factors contribute to larger infarct sizes, increased risk of HF, and higher mortality rates in diabetic patients with MI compared to their non-diabetic counterparts.

In recent years, the management of type 2 diabetes has undergone a paradigm shift with the introduction of sodium-glucose cotransporter 2 (SGLT2) inhibitors [5]. Among these, dapagliflozin has emerged as a promising agent with potential benefits extending beyond glycemic control. Dapagliflozin is a highly selective and reversible inhibitor of SGLT2, the primary glucose transporter in the proximal renal tubules. By inhibiting SGLT2, dapagliflozin reduces renal glucose reabsorption, leading to increased urinary glucose excretion and a consequent reduction in plasma glucose levels [6]. The mechanism of action of dapagliflozin offers several advantages in diabetes management. Unlike many traditional antidiabetic agents, SGLT2 inhibitors work independently of insulin, making them effective across various stages of diabetes progression. Additionally, the glucosuric effect of dapagliflozin is associated with caloric loss, contributing to weight reduction and improved insulin sensitivity. These properties, combined with its favorable safety profile, have positioned dapagliflozin as an important therapeutic option in the management of type 2 diabetes [5,6].

However, the impact of dapagliflozin extends beyond its glucose-lowering effects. Accumulating evidence suggests that SGLT2 inhibitors, including dapagliflozin, may confer cardiovascular benefits, particularly in patients with established cardiovascular disease or at high cardiovascular risk [7]. The potential cardioprotective mechanisms of dapagliflozin are multifaceted and include improvements in cardiac energy metabolism, reduction in preload and afterload, attenuation of cardiac fibrosis, and modulation of the renin-angiotensin-aldosterone system [7]. Given the high burden of HF and acute MI in diabetic patients, exploring the effects of dapagliflozin on these outcomes is of paramount importance. Understanding the impact of dapagliflozin on cardiovascular events and outcomes in this high-risk population could have significant implications for clinical practice and patient care. Moreover, elucidating the potential mechanisms by which dapagliflozin may influence HF and MI could provide valuable insights into novel therapeutic strategies for cardiovascular protection in diabetes.

The objectives of this systematic review are to evaluate the efficacy of dapagliflozin in reducing the incidence and progression of HF in diabetic patients, assess its impact on outcomes following MI in this population, and examine the potential mechanisms by which dapagliflozin may exert cardioprotective effects in the context of HF and MI. Additionally, the review aims to analyze the safety profile of dapagliflozin in diabetic patients with or at risk for HF and MI and to identify gaps in current knowledge, proposing directions for future research on the cardiovascular effects of dapagliflozin in diabetic patients. By systematically reviewing and synthesizing the available evidence, this review seeks to provide a comprehensive understanding of the role of dapagliflozin in HF and MI among diabetic patients, thereby informing clinical decision-making and guiding future research endeavors in this critical area of cardiovascular medicine.

## Review

Materials and methods

This systematic review is reported in accordance with the PRISMA (Preferred Reporting Items for Systematic Reviews and Meta-Analyses) guidelines to ensure a rigorous and comprehensive evaluation of the included studies on the effects of dapagliflozin on HF and acute MI in diabetic patients.

Search Strategy

A comprehensive search was conducted in the following databases from inception to May 2024: MEDLINE (via PubMed), Embase, Cochrane Central Register of Controlled Trials (CENTRAL), and Web of Science. The search strategy combined terms related to the population (e.g., "diabetes mellitus," "type 2 diabetes"), intervention (e.g., "dapagliflozin," "SGLT2 inhibitor"), and outcomes (e.g., "heart failure," "myocardial infarction," "cardiovascular outcomes"). We also performed hand-searching of the reference lists of relevant reviews and included studies to identify additional relevant publications that may not have been captured in the initial database search.

Eligibility Criteria

We included studies that met the following criteria: (1) the population consisted of adults with type 2 diabetes mellitus, irrespective of whether they had established cardiovascular disease; (2) the intervention was dapagliflozin, used either as a monotherapy or in combination with other antidiabetic agents; (3) comparators included placebo, no treatment, or other antidiabetic agents, but excluded other SGLT2 inhibitors; (4) primary outcomes focused on the incidence of HF events, such as new-onset HF or hospitalization due to HF, as well as outcomes following acute MI, including mortality, recurrent AMI, and HF; (5) secondary outcomes included changes in cardiac function parameters (e.g., ejection fraction, NT-proBNP levels), cardiovascular mortality, all-cause mortality, and safety outcomes such as hypoglycemia, ketoacidosis, and genital infections; and (6) study designs comprised randomized controlled trials (RCTs) and observational studies, including prospective and retrospective cohort studies, and case-control studies, with a minimum follow-up duration of 12 weeks. We excluded animal studies, in vitro experiments, reviews, editorials, conference abstracts, and studies not published in English. Case reports and case series were included only if they described rare adverse events.

Study Selection

Two independent reviewers conducted the study selection process. Initially, titles and abstracts of all retrieved records were screened to exclude clearly irrelevant studies. Full-text articles were then obtained for all potentially eligible studies and reviewed independently by both reviewers. Any discrepancies in study inclusion were resolved through discussion, and if necessary, a third reviewer was consulted to reach consensus.

Data Extraction

Data were extracted independently by two reviewers using a predesigned, piloted form in Microsoft Excel (Microsoft® Corp., Redmond, WA), focusing on several key aspects: study characteristics, including the author, year of publication, country, design, setting, and duration of follow-up; participant demographics, such as age, sex, duration of diabetes, baseline HbA1c levels, and the presence of cardiovascular comorbidities; details of the intervention and any co-interventions; specifics of the comparator; outcomes and main findings. Any disagreements that arose during data extraction were resolved through discussion between the reviewers or by consulting a third reviewer.

Data Synthesis and Analysis

Given the anticipated heterogeneity in study designs, patient populations, and outcome measures, we planned a primarily narrative synthesis of the findings. The results were summarized in descriptive tables, highlighting key characteristics and outcomes of each study. We performed a qualitative analysis to integrate and discuss the outcomes in the context of current clinical practice and future research directions.

This comprehensive methodology ensures a rigorous, transparent evaluation of the effects of dapagliflozin on HF and acute MI in diabetic patients, providing a strong evidence base to guide clinical decision-making and future research directions.

Results

Study Selection Process

**Figure 1:**
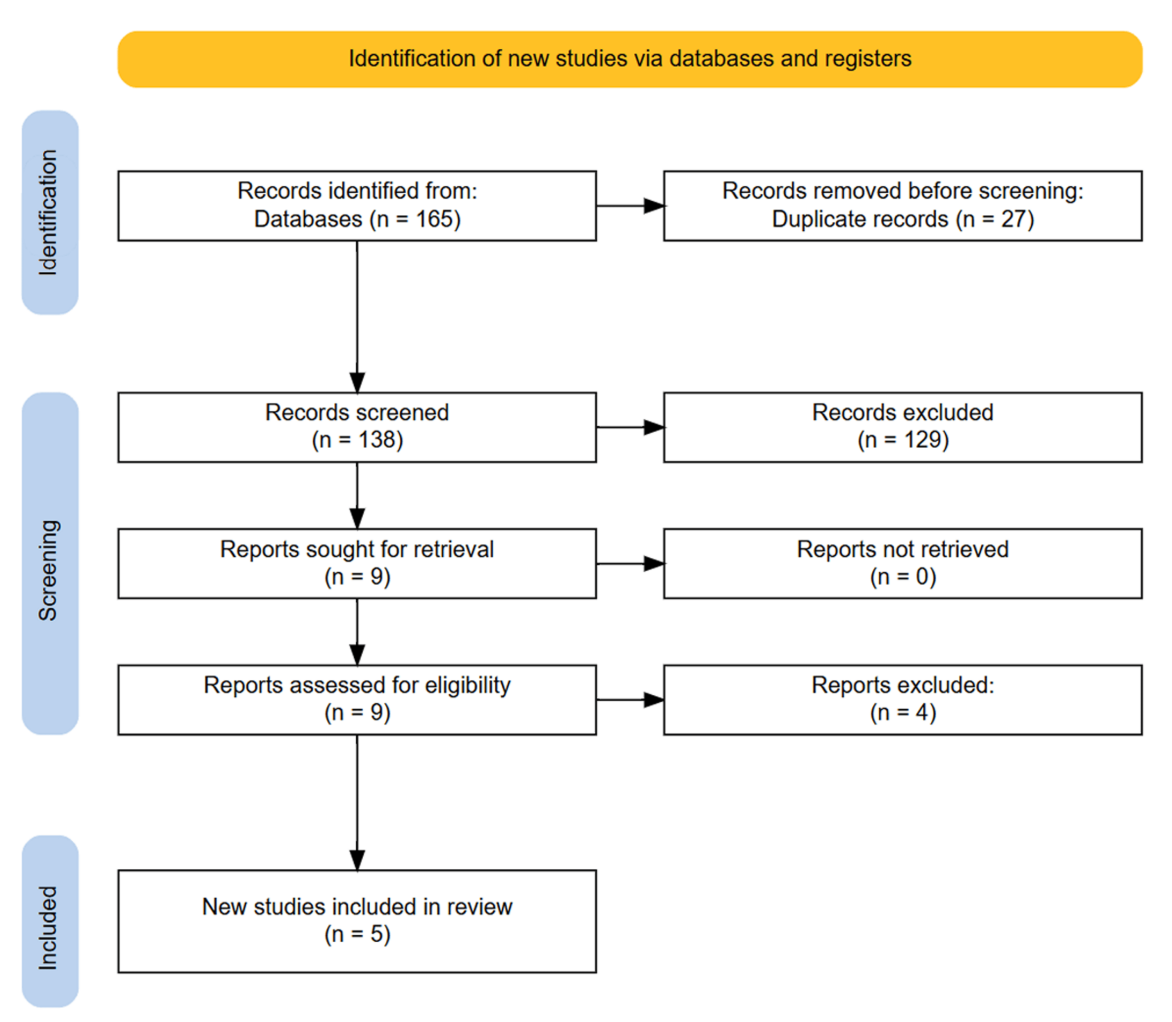
PRISMA diagram depicting the study selection process.

The initial database searches yielded 165 articles. After removing 27 duplicates, 138 publications underwent title and abstract screening. Subsequently, nine potential studies were assessed for eligibility through full-text examination. Five articles ultimately met the inclusion criteria. The reference lists of selected articles did not yield any additional eligible studies. PRISMA flowchart depicting the entire selection process is as follows (Figure 1).

Study Characteristics

The systematic review included five studies investigating the effects of dapagliflozin in T2DM patients with high cardiovascular risk. The studies were published between 2019 and 2023. Three of the five studies were RCTs, while two were retrospective analyses. The study duration varied considerably, ranging from 12 weeks to a median follow-up of 4.2 years [8,9]. The study by McMurray et al. had a median follow-up of 18.2 months, while the two retrospective studies by Mao et al. and Zhu et al. had median follow-ups of 540 days and 23 months, respectively [10-12].

The sample sizes ranged from 587 patients in the Nassif et al. study to 17,160 patients in the large-scale trial by Wiviott et al. [9]. The mean or median age of participants across studies ranged from the early 60s to the late 60s. Most studies reported a higher proportion of male participants, with percentages ranging from 57% to 76.8%. Baseline HbA1c levels varied across studies, reflecting the different patient populations. For instance, Wiviott et al. reported a mean HbA1c of 8.3% ± 1.2%, while Nassif et al. reported a median of 6.3% (IQR 5.6-7.6) [8,9]. Cardiovascular comorbidities were common, with high rates of hypertension, prior HF, and established atherosclerotic cardiovascular disease reported in several studies. All studies used dapagliflozin at a dose of 10 mg once daily. The duration of treatment ranged from 12 weeks to several years. Co-interventions typically included standard care for the respective conditions, such as other glucose-lowering drugs, standard HF therapies, or acute MI management in patients undergoing PCI.

The studies assessed a range of outcomes, with a focus on cardiovascular events, HF symptoms, and mortality. Common primary outcomes included major adverse cardiovascular events (MACE), HF hospitalizations, and composite endpoints of cardiovascular death or worsening HF. Secondary outcomes often included all-cause mortality, renal outcomes, and quality of life measures such as Kansas City Cardiomyopathy Questionnaire (KCCQ) scores. The main findings of the included studies are summarized in Table 1.

**Table 1:** Summary of the main findings of the included studies. MI: myocardial infarction, T2DM: type 2 diabetes mellitus, HF: heart failure, EF: ejection fraction, PCI: percutaneous coronary intervention, CV: cardiovascular, MACE: major adverse cardiovascular events, ACE: angiotensin-converting enzyme, DPP4: dipeptidyl peptidase-4, SGLT2: sodium-glucose cotransporter-2, KCCQ: Kansas City Cardiomyopathy Questionnaire, IV: intravenous, ARNI: angiotensin receptor-neprilysin inhibitor, HR: hazard ratio, CI: confidence interval, IQR: interquartile range.

Authors	Mao et al. [10]	Nassif et al. [8]	Zhu et al. [11]	McMurray et al. [12]	Wiviott et al. [9]
Publication year	2023	2023	2022	2019	2019
Journal	European Journal of Clinical Pharmacology	Circulation: heart failure	Cardiovascular Diabetology	New England Journal of Medicine	New England Journal of Medicine
Country	China	USA	China	Multinational (20 countries)	Multinational (33 countries)
Study design	Retrospective cohort	Randomized control trial	Retrospective analysis	Randomized control trial	Randomized control trial
Study duration	Patients enrolled between January 2017 and January 2021; median follow-up of 540 days	12 weeks	Patients enrolled between January 2019 and August 2021, with median follow-up of 23 months	Median follow-up of 18.2 months	Median follow-up of 4.2 years
Sample size	961 patients (275 in dapagliflozin group, 686 in non-dapagliflozin group)	587 patients (293 dapagliflozin, 294 placebo)	786 patients (645 in DAPA-free group, 141 in DAPA group)	4744 patients	17,160 patients
Population	Patients with acute MI and T2DM	Patients with HF across the full range of EF	Patients with acute MI undergoing PCI	Patients with HF and a reduced ejection fraction (≤40%)	Patients with T2DM who had or were at risk for atherosclerotic cardiovascular disease
Age	Dapagliflozin group: 61.97 ± 13.22 years; non-dapagliflozin group: 67.22 ± 12.15 years	Median 67 years (IQR 58-74)	Mean 62 ± 14 years overall	Not explicitly stated for overall population	Not specifically stated, but eligibility criteria included patients 40 years or older
Sex	Dapagliflozin group: 76.0% male; non-dapagliflozin group: 65.7% male	57% male overall	76.8% male overall	Not explicitly stated for overall population	Not explicitly stated for overall population
Baseline HbA1c	Dapagliflozin group: 8.10 [7.10, 9.50]%; non-dapagliflozin group: 7.60 [6.60, 8.78]%	Median 6.3% (IQR 5.6-7.6)	6.2 ± 1.2% in DAPA-free group, 7.9 ± 1.7% in DAPA group	Not explicitly stated	Mean 8.3% ± 1.2%
Baseline CV comorbidities	Hypertension: 77.8% in dapagliflozin group, 78.4% in non-dapagliflozin group	66% prior HF hospitalization, 34% ischemic heart disease, 59% T2DM, 47% atrial fibrillation	Higher rates of hypertension and diabetes in DAPA group	All patients had HF with reduced ejection fraction	40.6% had established atherosclerotic cardiovascular disease, 10% had history of HF
Dapagliflozin dose	10 mg once daily	10 mg once daily	Not specified	10 mg once daily	10 mg once daily
Duration of treatment	In-hospital and continued use after discharge	12 weeks	Patients received dapagliflozin at discharge and were followed for median 23 months	Median 18.2 months	Median 4.2 years
Co-intervention	Other glucose-lowering drugs (including sulfonylureas, glinides, α-glucosidase inhibitors, DPP4 inhibitors, insulin)	Standard HF therapies (e.g. ACE inhibitors, beta blockers, diuretics)	Standard care for acute MI patients undergoing PCI	Standard HF therapies	Other glucose-lowering agents allowed at discretion of treating physician (except open-label SGLT2 inhibitors, pioglitazone, or rosiglitazone)
Outcomes	Primary outcome: incidence of HF rehospitalization; secondary outcomes: adverse drug events (volume depletion, hyperkalemia, ketoacidosis), all-cause mortality	Change in KCCQ scores at 12 weeks	Primary endpoint was composite of MACE, including overall deaths, HF, nonfatal MI, nonfatal stroke, and unplanned repeat revascularization	Primary composite outcome: Worsening HF (hospitalization or urgent visit requiring IV therapy) or cardiovascular death; Secondary outcomes included hospitalization for HF, cardiovascular death, KCCQ score, renal composite outcome, all-cause mortality	Primary safety outcome: MACE; Primary efficacy outcomes: 1) MACE, 2) Composite of cardiovascular death or hospitalization for HF; Secondary outcomes: Renal composite outcome, all-cause mortality
Results	The dapagliflozin group had a significantly lower HF rehospitalization rate (6.9% vs. 16.5%, p<0.001) and reduced risk of rehospitalization (HR = 0.498, 95% CI = 0.296-0.839, p=0.008). These findings were consistent across sensitivity and subgroup analyses, with a low incidence of adverse events in the dapagliflozin group.	Dapagliflozin significantly improved the KCCQ Clinical Summary Score by 5.0 points (95% CI 2.6-7.5, p<0.001) compared to placebo, demonstrating benefits across the entire spectrum of EFs. Patients treated with dapagliflozin experienced less deterioration in KCCQ scores and a higher proportion achieved clinically meaningful improvements.	DAPA group exhibited a significantly lower incidence of MACE at 8.5% compared to 18.3% in the control group (p=0.018). Multivariate analysis revealed that DAPA was associated with a reduced risk of MACE, with HR of 0.388 (95% CI 0.192-0.785, p=0.008). Additionally, the DAPA group experienced lower rates of HF, nonfatal MI, and unplanned repeat revascularization. At 12 months, DAPA also led to reductions in the TyG index and the atherogenic index of plasma.	In the study, treatment with dapagliflozin resulted in a significantly lower incidence of the primary outcome compared to placebo (16.3% vs. 21.2%; HR 0.74, 95% CI 0.65-0.85, p<0.001). Specifically, rates of cardiovascular death (9.6% vs. 11.5%; HR 0.82, 95% CI 0.69-0.98) and all-cause mortality (11.6% vs. 13.9%; HR 0.83, 95% CI 0.71-0.97) were also reduced with dapagliflozin. Patients reported improved symptoms based on the KCCQ, with consistent benefits observed regardless of diabetes status.	Dapagliflozin demonstrated non-inferiority to placebo in MACE. It significantly reduced the risk of cardiovascular death or hospitalization for HF compared to placebo (4.9% vs 5.8%, HR 0.83, p=0.005), but did not show a significant difference in overall MACE incidence (8.8% vs 9.4%, HR 0.93, p=0.17). Dapagliflozin also exhibited a reduction in renal composite outcomes (4.3% vs 5.6%, HR 0.76), while all-cause mortality rates between dapagliflozin and placebo groups were not significantly different (6.2% vs 6.6%, HR 0.93).
Summary of the study	In patients with diabetic acute MI, the in-hospital and continued use of dapagliflozin after discharge was linked to a notably lower risk of HF rehospitalization compared to those not using dapagliflozin. The study indicates significant benefits of early dapagliflozin administration in this high-risk group.	n patients with HF across the full spectrum of EF, dapagliflozin significantly improved symptoms and physical limitations after 12 weeks of treatment, with consistent benefits regardless of baseline EF. The study provides evidence supporting the use of dapagliflozin to improve health status in HF patients across the range of EF.	In patients with acute MI undergoing PCI, dapagliflozin treatment was associated with significantly lower risk of MACE and improvements in metabolic parameters over 23 months follow-up. The protective effect was most pronounced in older patients, those with hypertension, and those not receiving ARNI therapy.	In patients with HF and reduced ejection fraction, dapagliflozin reduced the risk of worsening HF or cardiovascular death compared to placebo, regardless of diabetes status. It also improved HF symptoms and reduced all-cause mortality.	In patients with type 2 diabetes at high cardiovascular risk, dapagliflozin was non-inferior to placebo for MACE. It reduced the composite of cardiovascular death or hospitalization for HF, primarily driven by reduced HF hospitalizations. Dapagliflozin also reduced renal events but did not significantly reduce MACE or all-cause mortality compared to placebo.

Discussion

The studies consistently demonstrate the beneficial effects of dapagliflozin on cardiovascular outcomes, particularly in relation to HF. The large-scale trial by Wiviott et al. showed that dapagliflozin significantly reduced the composite of cardiovascular death or hospitalization for HF compared to placebo (4.9% vs. 5.8%, HR 0.83, p=0.005) [9]. This finding is particularly noteworthy given the high-risk population studied, which included patients with T2DM who had or were at risk for atherosclerotic cardiovascular disease. McMurray et al. specifically focused on patients with HF and reduced ejection fraction (HFrEF), demonstrating that dapagliflozin significantly lowered the incidence of worsening HF or cardiovascular death compared to placebo (16.3% vs. 21.2%; HR 0.74, 95% CI 0.65-0.85, p<0.001) [12]. Importantly, this benefit was observed regardless of diabetes status, suggesting that the cardioprotective effects of dapagliflozin extend beyond its glucose-lowering properties. The retrospective study by Mao et al. corroborated these findings in a real-world setting, showing a significantly lower HF rehospitalization rate in diabetic acute MI patients treated with dapagliflozin (6.9% vs. 16.5%, p<0.001) [10]. This study highlights the potential benefits of early dapagliflozin administration in high-risk patients with acute MI. These results collectively suggest that dapagliflozin may play a crucial role in preventing and managing HF in diabetic patients, both in those with established HF and in those at risk for developing HF. The consistency of these findings across different study designs and patient populations strengthens the evidence for the cardioprotective effects of dapagliflozin.

While the primary focus of most studies was on HF outcomes, the review also provides insights into the effects of dapagliflozin on acute MI. The retrospective analysis by Zhu et al. specifically examined patients with acute MI undergoing PCI [11]. They found that dapagliflozin treatment was associated with a significantly lower incidence of MACE at 8.5% compared to 18.3% in the control group (p=0.018). This study suggests that dapagliflozin may offer protective effects in the acute post-MI setting, potentially reducing the risk of recurrent events and improving overall outcomes. However, it is important to note that Wiviott et al. did not show a significant difference in overall MACE incidence between dapagliflozin and placebo groups (8.8% vs. 9.4%, HR 0.93, p=0.17) [9]. This discrepancy highlights the need for further research to clarify the specific effects of dapagliflozin on acute MI outcomes and to identify which subgroups of patients may derive the greatest benefit.

The cardiovascular benefits of dapagliflozin appear to result from a combination of multiple mechanisms. While the specific studies reviewed did not directly examine these mechanisms, prior research, and the documented outcomes suggest several plausible pathways. First, the hemodynamic effects of dapagliflozin, due to its diuretic and natriuretic properties, may lower both preload and afterload, thus enhancing cardiac function [13,14]. Second, the capacity of this drug to reduce glucose levels and enhance insulin sensitivity may lead to improved myocardial energy metabolism [15]. Additionally, dapagliflozin might have anti-inflammatory and anti-fibrotic effects that reduce cardiac inflammation and fibrosis, thereby preserving myocardial structure and function [16]. Furthermore, there is evidence to suggest that SGLT2 inhibitors, like dapagliflozin, may exert direct beneficial effects on cardiomyocytes, enhancing their function and survival. Lastly, the renoprotective effects of dapagliflozin, as shown by the favorable renal outcomes by Wiviott et al., could indirectly support cardiovascular health through improved cardiorenal interactions [9].

An important aspect of HF management is improving the quality of life and symptom burden of patients. The study by Nassif et al. specifically addressed this, showing that dapagliflozin significantly improved the KCCQ clinical summary score by 5.0 points (95% CI 2.6-7.5, p<0.001) compared to placebo [8]. This improvement was consistent across the entire spectrum of ejection fractions, suggesting that dapagliflozin may benefit patients with HF regardless of their left ventricular function. McMurray et al. also reported improvements in HF symptoms based on the KCCQ, with consistent benefits observed regardless of diabetes status [12]. These findings underscore the potential of dapagliflozin to not only reduce hard clinical endpoints but also to improve patients' subjective experience of their disease, which is a crucial aspect of comprehensive HF management.

The safety profile of dapagliflozin in the context of HF and MI is an important consideration. Across the studies, dapagliflozin was generally well-tolerated. Mao et al. reported a low incidence of adverse events in the dapagliflozin group, which is reassuring given the high-risk nature of the acute MI population studied [10]. However, it is important to note that SGLT2 inhibitors, including dapagliflozin, have been associated with certain adverse effects, such as genital infections, volume depletion, and rare cases of diabetic ketoacidosis [17-19]. While these were not prominently reported in the studies included in this review, clinicians should remain vigilant and monitor patients appropriately when initiating dapagliflozin treatment, especially in vulnerable populations such as elderly patients or those with pre-existing renal impairment [19,20].

The findings of this systematic review highlight several critical implications for clinical practice. First, dapagliflozin emerges as a valuable treatment option for patients with T2DM who either have HF or are at high risk for it due to its consistent benefits in reducing HF hospitalizations and improving cardiovascular outcomes [9,12,21]. Additionally, the benefits of dapagliflozin in patients with HFrEF extend to those without diabetes, indicating that this medication could play a significant role in HF management irrespective of diabetes status. Importantly, the early initiation of dapagliflozin in high-risk patients, such as those experiencing an acute MI, may offer substantial cardiovascular protection, making it a valuable component of comprehensive post-MI care [10]. Furthermore, the notable improvement in quality of life and symptom burden among HF patients using dapagliflozin suggests that this medication provides benefits beyond conventional clinical endpoints [8]. Despite its generally favorable tolerability profile, careful patient selection and monitoring are crucial when initiating dapagliflozin treatment, particularly in patients with multiple comorbidities or those at increased risk for adverse effects.

Despite the valuable insights provided by this systematic review, several limitations and areas for future research warrant attention. First, the heterogeneity in study designs and populations, encompassing both randomized controlled trials and retrospective analyses, introduces variability in the quality and interpretation of evidence. Future meta-analyses could help quantify the overall effect size of dapagliflozin on various outcomes and provide a more cohesive understanding of its impact. Second, while some studies included in the review had extended follow-up periods, there is a need for more research to explore the long-term effects of dapagliflozin on cardiovascular outcomes, particularly in patients with HF. Third, the precise mechanisms through which dapagliflozin exerts its cardioprotective effects, especially in the context of acute MI, remain to be fully elucidated, necessitating further mechanistic studies. Additionally, more detailed subgroup analyses are needed to identify which specific patient populations, based on HF etiology, renal function, or concomitant medications, are most likely to benefit from dapagliflozin treatment. Comparative effectiveness studies that assess dapagliflozin against other SGLT2 inhibitors and glucose-lowering medications in terms of cardiovascular protection would also be valuable. Lastly, evaluating the cost-effectiveness of dapagliflozin in reducing HF hospitalizations and improving cardiovascular outcomes could provide important insights for healthcare policy and resource allocation, guiding more efficient and effective treatment strategies [22].

In conclusion, this systematic review provides compelling evidence for the cardiovascular benefits of dapagliflozin in patients with T2DM, particularly in the context of HF and, to some extent, acute MI. The consistent reduction in HF hospitalizations, improvement in quality of life, and potential benefits in post-MI care position dapagliflozin as an important therapeutic option in the management of high-risk diabetic patients with cardiovascular disease. Future research should focus on refining our understanding of the mechanisms of action, identifying optimal patient populations, and evaluating long-term outcomes to further optimize the use of dapagliflozin in clinical practice.

## Conclusions

There is compelling evidence for the cardiovascular benefits of dapagliflozin in patients with type 2 diabetes mellitus, particularly in the context of heart failure. The consistent reduction in HF hospitalizations, improvement in quality of life, and potential benefits in post-MI care position dapagliflozin as an important therapeutic option for high-risk diabetic patients with cardiovascular disease. The cardioprotective effects appear to extend beyond glycemic control, suggesting a multifaceted mechanism of action. While the evidence for benefits in acute MI is less conclusive, the overall safety profile and positive impact on HF outcomes make dapagliflozin a valuable addition to the management of diabetic patients with or at risk for cardiovascular complications. Future research should focus on elucidating the precise mechanisms of action, identifying optimal patient populations, and evaluating long-term outcomes. Additionally, comparative effectiveness studies and cost-effectiveness analyses could further guide clinical decision-making and resource allocation in the use of dapagliflozin for cardiovascular protection in diabetic patients.
